# Homophobic beliefs and attitudes among mid-adolescent boys: exploring the ideas of hybrid masculinities

**DOI:** 10.3389/fsoc.2024.1347568

**Published:** 2024-06-11

**Authors:** Deinera Exner-Cortens, Caroline Claussen, Angelique Jenney, Vineetha Warriyar Kodalore Vijayan

**Affiliations:** ^1^Department of Psychology, University of Calgary, Calgary, AB, Canada; ^2^Faculty of Social Work, University of Calgary, Calgary, AB, Canada; ^3^Alberta Children’s Hospital Research Institute (ACHRI), University of Calgary, Calgary, AB, Canada

**Keywords:** adolescents, hybrid masculinities, homophobia, male role norms, gender policing

## Abstract

**Introduction:**

Homophobia is well-documented as key to social regulation of masculine behavior and practices in Western settings. Yet, empirical data from a number of Western settings has shown a decline in overt homophobic attitudes in the past decade, leading some to suggest that the nature of masculinities is also changing. However, theorizing on the changing nature of masculinities among adolescents has received limited quantitative attention. Research is needed to better understand shifts in adolescent masculinities in contemporary Western settings.

**Methods:**

In this paper, we investigate the application of one newer approach to explore masculinities in context – hybrid masculinities – in a sample of cisgender, heterosexual, mid-adolescent boys in one province in Western Canada (*N =* 873, mean age (SD) = 14.39 (0.37)). Data were collected from nine cohorts of grade 9 youth over a 10-year period (2013–2022) as part of the baseline survey of an ongoing evaluation of a gender-transformative healthy relationships program.

**Results and discussion:**

We hypothesized that if the ideas of hybrid masculinities held in our sample, we would find that overt homophobic attitudes and adherence to related patriarchal norms (e.g., avoidance of femininity) would decline over this period, but that the use of homophobic name-calling would remain differentiated in terms of to whom it was directed (e.g., a friend, someone they thought was gay). We did find a significant decline in homophobic attitudes and norms related to emotional restriction and avoidance of femininity over the 10-year period, but also found that homophobic name-calling remained differentiated, with significantly higher name-calling toward a friend than toward someone the youth thought was gay. Thus, our hypotheses were supported. We discuss the implications of our findings for future theory and research on understanding adolescent masculinities in context.

## 1 Introduction

The concept of hegemonic masculinity has shaped a substantial amount of literature on men and masculinities in the past several decades (Hearn, 2004; Connell and Messerschmidt, 2005; Messerschmidt, 2012). First proposed by Connell (1987), and re-specified by Connell and Messerschmidt (2005), this construct proposes that there are hierarchical relationships among various masculinities, that this hierarchy is supported by hegemony (e.g., cultural sanctioning, institutionalization), and that some of the masculinities within this hierarchy (locally, regionally, or globally) are more socially powerful than others. The enactment of hegemonic masculinity thus maintains continued inequality, both between and within genders. Per its hierarchical nature, the hegemonic framework also orders specific relations of dominance and subordination between groups of men, with gay masculinities traditionally being at the bottom of the male gender hierarchy (Connell, 2005).

Maintaining hegemony requires active gender policing of men by men, in addition to the continued exclusion of women and other marginalized genders (Connell and Messerschmidt, 2005). As defined by Reigeluth and Addis (2015), policing of masculinity consists of “any action that serves to prevent or punish individual or group behavior perceived as insufficiently masculine” (p. 75), and is a process which supports gendered social learning. This policing is often done through homophobic name-calling (Pascoe, 2007; Gough et al., 2021). Indeed, it is through such policing practices that homophobic name-calling has been understood as a tool through which hegemonic masculinity is constituted and maintained (Pascoe, 2007; Bridges and Pascoe, 2016).

Although homophobic name-calling (and homophobia in general) have long been considered central tenets of adolescent masculinity in the West (Pascoe, 2007; Bridges and Pascoe, 2014; Birkett and Espelage, 2015), the past decade has seen societal changes with regard to improving attitudes toward homosexuality broadly (i.e., more overtly accepting; Flores, 2014; Twenge et al., 2016; Pew Research Center, 2019). These shifts seem to challenge understandings of homophobia as a core feature of hegemonic masculinity in the Western context, and the related subordination of gay masculinities (Anderson, 2009, 2013; Anderson and McGuire, 2010; McCormack, 2012; Anderson and McCormack, 2018).

To this end, more recent research in the field has explored the diminishing impact of homophobia on adolescent masculinities, suggesting the theoretical presence of multiple forms of masculinity, potentially *without* hegemonic dominance of any one over the other (Dashper, 2012; McCormack, 2012; Anderson, 2013; McCormack and Anderson, 2014a,b). Contemporary literature identifies different streams of research that account for the apparently changing nature of masculinities among adolescents. Inclusive Masculinity Theory (Anderson, 2013; Anderson and McCormack, 2018) and hybrid masculinities (Bridges, 2014, 2021; Bridges and Pascoe, 2014) are two streams that both suggest changes to masculinities are occurring, although they differ in terms of whether the change is indicative of larger changes in gender and sexuality equality. Our investigation examines the applicability of one of these accounts, hybrid masculinities, by exploring quantitative data collected with 873 adolescent boys from 2013 to 2022 in one Western setting.

Research on men and masculinities has undergone shifts in focus over the past several decades, in response to both changing accounts of and within masculinities over time (Smiler, 2004; Messerschmidt and Messner, 2018). Early work explored masculine gender role stress (Eisler and Skidmore, 1977), gender role conflict (O’Neil, 2008) and gender role strain (Levant, 2011), all representing different conceptualizations of stress and coping arising from performing the male gender role in Western contexts (e.g., emotional suppression). Building on these explorations, more recent work has explored the precarious nature of “manhood” status (Vandello and Bosson, 2013); different norms associated with non-dominant masculinities (e.g., Spencer et al., 2004; Smiler, 2006); and changes to structural arrangements of masculinity in Western contexts (McCormack, 2012; Anderson, 2013; Bridges, 2014; Bridges and Pascoe, 2018). To add to this conversation, in this article, we focus on further exploration of one of these more contemporary accounts: hybrid masculinities.

The concept of hybrid (hegemonic) masculinities acknowledges that while contemporary masculinities may (or may appear to be) changing, existing ideologies and power relations are less challenged than some other theories posit (Bridges and Pascoe, 2014, 2018; Bridges, 2021). Specifically, hybrid masculinities scholarship focuses on the ways in which certain men – typically privileged, young, heterosexual, White men – may selectively incorporate elements of marginalized masculinities and/or femininities into their gendered practices and identities (Bridges, 2014, 2021; Bridges and Pascoe, 2014; Pfaffendorf, 2017; Munsch and Gruys, 2018). As noted by Bridges (2021), “hybrid masculinities refers broadly to a collection of masculine gender projects that incorporate elements of identity socially and culturally associated with ‘others’…research and theory on hybrid masculinity seek to understand gender practices that blur social differences while simultaneously considering their relationships with different axes of social *dominance*” (p. 665). Accordingly, privileged young men may espouse politically progressive attitudes while simultaneously fortifying “existing symbolic boundaries that conceal systems of power and inequality in historically new ways” (Bridges and Pascoe, 2014, p. 246).

Research on hybrid masculinities points to several consequences of shifting gender projects and performance that potentially exacerbate, reflect, and conceal inequalities (Bridges and Pascoe, 2018). The first consequence is what Bridges and Pascoe (2014) call “discursive distancing,” which refers to participation in practices that allow men to distance themselves from the hegemonic form of masculinity, while simultaneously continuing to perpetuate patriarchal norms and beliefs (hooks, 2004). Bridges (2010) gives the example of Walk A Mile in Her Shoes events, which are designed to bring awareness to domestic and sexual violence through men’s active participation. In these walks, men wear high heels to symbolically represent how difficult it is to walk in “her shoes.” This event is designed to show men’s solidarity with a feminist cause, and while men who participate may learn about women’s experiences, participation can also contribute to a reification of gendered norms and practices (e.g., wearing high heels; joking about acting ‘like a woman’; Bridges, 2010). In sum, discursive distancing is a particular “hybrid hegemonic tactic that positions men in ways that simultaneously secures and obscures their relationships with enduring systems of gendered and racialized power and control” (Bridges, 2021, p. 675).

The second consequence discussed by Bridges and Pascoe (2014) is “strategic borrowing,” which refers to the appropriation of aspects of marginalized masculinities (particularly, gay and racialized masculinities) by White, heterosexual young men. In other words, heterosexual young men may incorporate aspects of these ‘othered’ masculinities (e.g., emotional intimacy and sensitivity) into their performance and practices, but without explicitly challenging systems of gender and/or sexual inequality (Eisen and Yamashita, 2019; Christofidou, 2021). For example, some men may adopt practices of emotional sensitivity to be more sexually successful with women. In this case, these men are appropriating aspects of feminized practices of masculinity to enhance their chance of achieving a hegemonic goal, and not because they are working to fundamentally disrupt patriarchal masculinity (hooks, 2004), and thus this is an example of strategic borrowing.

The third consequence discussed by Bridges and Pascoe (2014) is “fortifying boundaries.” Specifically, through discursive distancing and strategic borrowing, the boundaries between more and less powerful masculinities become strengthened, because strategic borrowing “obscur[es] the symbolic and social boundaries between groups upon which such practices rely” (Bridges and Pascoe, 2014, p. 254). In other words, because discursive distancing and strategic borrowing only change the appearance of masculinities on the surface – and not the underlying structure of power and privilege – boundaries between dominant and marginalized masculinities remain unchanged, and due to the now more invisible nature of these boundaries, fortified.

### 1.1 Current study

A hybrid masculinities lens holds promise for our understanding of adolescent masculinities in modern Western context. However, there is little research that has quantitatively explored the application of this concept, particularly with adolescent samples (Connor et al., 2021). As noted by Mittleman (2023), “despite ethnographic evidence…the invisibility of gender expression in most social surveys has made gender policing difficult to document” (p. 6). There have been some studies of adult populations that have found a relationship between masculinity and homophobia when examining attitudes related to same-sex couples and romantic behaviors (Doan et al., 2014; Mize and Manago, 2018). More recently, Mittleman (2023) used population-based data from a geographically diverse adolescent sample to examine bullying as a form of gender policing, finding that gender expression significantly influenced and shaped bullying victimization for cisgender, heterosexual boys. In this paper, we build on this limited body of research by investigating the application of the ideas of hybrid masculinities with a sample of cisgender, heterosexual, mid-adolescent boys in one province in Western Canada. We explore change over time in overt homophobic attitudes and adherence to related patriarchal norms, as well as the directionality of homophobic name-calling, over a ten-year period.

As it pertains to homophobia and its role in constituting powerful masculinities, a hybrid masculinities approach argues that, while on the surface non-homophobic masculinities appear to be proliferating, the continued use of homophobic name-calling, particularly among peers, suggests the relationships between dominant forms of adolescent masculinity and homophobia may still be entrenched (Bridges and Pascoe, 2014, 2018). Per the three theoretical consequences of hybrid masculinities described above, we would expect to see an overall decline in overt homophobic attitudes and related patriarchal norms (e.g., emotional restriction, avoidance of femininity, toughness) over time, as young men engage in discursive distancing (e.g., stating explicit disagreement with fear of gay men) and strategic borrowing (e.g., assimilating aspects of feminized masculinities, like more emotional intimacy). But, because these changes do not touch the underlying structure of the hegemonic form of masculinity, we also anticipate that boys will continue to use homophobic name-calling, that the overall use of this name-calling will not be strongly associated with reported homophobic attitudes, and that this name-calling will be differentiated (e.g., much more likely to use with a friend than someone they actually perceive to be gay), in their efforts to fortify boundaries between dominant and marginalized masculinities.

As part of the baseline survey of an evaluation of a gender-transformative healthy relationships program for adolescent boys (Exner-Cortens et al., 2019, 2020), we collected data on overt homophobic attitudes and adherence to related patriarchal norms from fall 2013-fall 2022 (*N* = 873), and data on homophobic name-calling from fall 2016-fall 2022 (*n* = 562). These data allow us to quantitatively explore the ideas of hybrid masculinities (Bridges and Pascoe, 2014). Specifically, we hypothesize that, if the ideas of hybrid masculinities apply in our sample, (1) overt homophobic attitudes and adherence to related patriarchal norms will decline over time, but that (2) (a) use of overall homophobic name-calling and name-calling by target (e.g., friend vs. someone they thought was gay) will have small-to-moderate correlations with self-reported homophobic attitudes and (b) since homophobic name-calling is used in a way to fortify boundaries (i.e., identifying outsiders in the group and reproducing social inequalities; Pascoe, 2013; Bridges and Pascoe, 2018), homophobic name-calling will be differentiated by the person it is directed at, with the most common use being toward a friend (Reigeluth and Addis, 2015).

We will explore these hypotheses in the full sample, and in a sub-sample identified as important in previous research and theorizing on adolescent masculinities: Ethnocultural boys. By Ethnocultural boys, we mean “non-White groups in Canada that have a distinct cultural, ethnic and linguistic heritage… This term recognizes and acknowledges that their culture is diverse from what is considered dominant in Canada. While these youth face racialization…and associated barriers, they also show resilience in the face of continued systemic racism” (Exner-Cortens et al., 2022, p. 6). We include this group as the interplay of gender and race in settler-colonial societies like Canada results in marginalized masculinities (Connell, 2005). Marginalized masculinities share some ground with hegemonic forms of masculinity but are ultimately marginalized from the benefits of hegemony (Connell, 2005; Pascoe and Bridges, 2016). For example, because of the systemic racism they face across settings and institutions in Canada, Ethnocultural boys may feel stronger pressure than their White counterparts to adhere to stereotypical expectations of masculinity (Griffith et al., 2012; Exner-Cortens et al., 2022). It is also possible that hybrid gender identities are crafted differently by marginalized and subordinated groups (Bridges and Pascoe, 2014). Thus, it is important to specifically explore whether the ideas of hybrid masculinity also extend to specific a sub-group that has typically experienced marginalized masculinities in Western settings.

## 2 Methods

### 2.1 Participants and procedure

Participants for this study were drawn from the baseline (i.e., pre-test) data of an ongoing evaluation study of a gender-transformative healthy relationships program called WiseGuyz. All participants came from schools in and around a large metropolitan region in one province in Western Canada. WiseGuyz is a gender-transformative healthy relationships program for adolescent boys developed by the Centre for Sexuality that aims to improve mental and sexual health and reduce violence by deconstructing patriarchal gender norms (Exner-Cortens et al., 2019, 2020). From fall 2013-fall 2018, adolescents were eligible to participate in the research project if they were enrolled in the WiseGuyz program within the relevant academic year. From fall 2019-fall 2022, we expanded our evaluation to include grade 9 boys who were and were not in WiseGuyz, and so any grade 9 boy at a school were WiseGuyz was offered in that academic year was eligible to participate. We note that because of the pandemic, we were unable to collect data in fall 2020 (Supplementary Table S1). Thus, we have data from nine cohorts of grade 9 boys over a 10-year period. Sample size across years ranges from 15 to 142 (Supplementary Table S1). Adolescents join WiseGuyz voluntarily, or with the gentle encouragement of an administrator, parent or teacher; however, participation is always voluntary. All WiseGuyz participants (fall 2013-fall 2018)/grade 9 boys at a school were WiseGuyz was offered (fall 2019-fall 2022) are given the option to participate in research. All participants require signed parent/guardian consent per school division rules, and also provide youth assent prior to completing the survey. Baseline surveys are completed prior to the start of program content at a given school. Surveys over the study period took between 20 and 30 min to complete and were offered on paper and/or electronically during the school day. This research was reviewed and approved by a university Research Ethics Board, as well as the participating schools and school divisions.

### 2.2 Measures

#### 2.2.1 Homophobic attitudes

Were assessed using the Negativity Toward Sexual Minorities (NTSM) scale (Levant et al., 2010). The NTSM was administered on all baseline surveys from 2013 to 2022. This 9-item scale taps overt homophobic attitudes (e.g., “it is disappointing to learn that a famous athlete is gay”; *α* = 0.96). To make items more applicable in a Canadian context, we dropped one item from all surveys (“Gay people should not be allowed to serve in the military”), leaving us with an 8-item scale for analysis. We also changed all references to “homosexual” in the original scale to “gay people” in the version we used on our surveys. Responses were measured on a 7-point Likert-type scale (1 = *strongly disagree* to 7 = *strongly agree*). Scores were summed across all eight items to create the total score, with higher scores indicating more homophobic attitudes toward gay men. Psychometric evidence for the NTSM was originally obtained in a sample of undergraduates. However, in our data, NTSM scores correlate with MRNI-A-r scores as anticipated (*r* range = 0.45–0.69), providing evidence of convergent validity, and internal consistency reliability is strong.

#### 2.2.2 Adherence to patriarchal norms

Was assessed using the Male Role Norms Inventory-Adolescent-revised (MRNI-A-r; Levant et al., 2012). The MRNI-A-r was administered on all baseline surveys from 2013–2022. This 29-item scale taps three domains: Emotionally Detached Dominance (EDD; e.g., “guys should not ever show their feelings”; 16 items, *α* = 0.91); Avoidance of Femininity (AF; e.g., “guys should not carry purses”; 6 items, *α* = 0.85); and Toughness (T; e.g., “it’s important for a guy to be able to play it cool”; 7 items, *α* = 0.80). Responses were measured on a 7-point Likert-type scale (1 = *strongly disagree* to 7 = *strongly agree*). Scores were summed across items within each sub-scale, with higher scores indicating more adherence to patriarchal gender norms. The MRNI-A-r has evidence of reliability and validity with early adolescents (Levant et al., 2012).

#### 2.2.3 Homophobic name-calling

Was assessed using the Homophobic Content Agent Target scale (Poteat and Espelage, 2005). This scale assesses the use of homophobic name-calling (e.g., gay, lesbo, fag) toward others (agent) or that is experienced by the participant (target). In this study, we only administered the 5-item Agent sub-scale (Homophobic Content Agent; HCA), because of our interest in the use of homophobic language by program participants. Agents included on this scale were (1) a friend, (2) someone I did not know, (3) someone I did not like, (4) someone I thought was gay, and (5) someone I did not think was gay. This scale was only added to baseline surveys in 2016, and so data are only available for 2016–2022. Further, in 2016, we were testing multiple bullying scales, and so only a random third of participants were assigned this sub-scale. However, in fall 2017, fall 2019, fall 2021, and fall 2022, all participants completed this measure (Supplementary Table S1). Responses were measured on a 5-point Likert-type scale (1 = *never* to 5 = *7 or more times*). Scores were summed to create a Total score, and to create a score for each specific Agent; higher scores indicate more homophobic name-calling overall/toward the particular Agent. Based on response patterns, we also dichotomized responses for each Agent into 1 = *any use* and 0 = *no use* for analyses. The HCA has evidence of reliability and validity in a sample of grade 8 students (Poteat and Espelage, 2005).

#### 2.2.4 Demographics

In all years, we assessed age on the pre-test race/ethnicity using Statistics Canada categories [for bivariate and multivariable analyses, collapsed into White and Ethnocultural (African, East Asian, Filipino, Indigenous, Latin American, Middle Eastern/West Asian, South Asian, Southeast Asian, Other, Multi-Ethnic)], dating status (1 = *ever dated;* 0 = *no/not sure*), family structure (1 = *dual caregiver home*; 0 = *other family structure arrangement*), and gender. Across all years, we also assessed sexual orientation/attraction. For this variable, from fall 2013–2015, participants were asked how they identified (heterosexual, gay, bisexual, not sure, rather not say), while from 2016 to 2022, participants were asked about their sexual attraction using a Kinsey-type scale (from *only attracted to females* to *only attracted to males;* participants could also answer that they were not sure or that they were not attracted to anyone). For analyses, we combined these variables into the categories of *exclusively heterosexual* (i.e., identify as heterosexual or report they were only attracted to females) and *not exclusively heterosexual* (i.e., identify as gay/bisexual/not sure/rather not say or report they are not exclusively attracted to females/that they were not sure/that they were not attracted to anyone). For clarity, we refer to this composite variable as sexual orientation in this paper.

### 2.3 Analysis

From 2013 to 2022, we collected data from a total of 1,075 grade 9 boys (Supplementary Table S1). Because the ideas of hybrid masculinities primarily pertain to cisgender, heterosexual men and boys (Bridges and Pascoe, 2014), our analysis specifically includes participants who self-reported that they identified as cisgender, heterosexual boys (*N* = 873).

In terms of the sample, there were two key changes in data collection that we wanted to further explore prior to testing our hypotheses. First, as described above, from fall 2019-fall 2022, we began to collect data from grade 9 boys who both were and were not participating in WiseGuyz. Second, because of a change in grant funding, we expanded the number of school divisions with whom we collected data in fall 2018. Although we were still collecting data in the same metropolitan area, data from fall 2018-fall 2022 were collected from a mix of three urban, suburban, and rural divisions, while data from fall 2013-fall 2017 were collected in one urban school division only. Using independent samples t-tests, we thus explored whether there were differences in NTSM, MRNI-A-r, or HCA (total) scores by (1) WiseGuyz participation or (2) population center type of where the participant attended school (small, medium or large, based on 2021 Canadian Census data).

In the Results, we present demographic characteristics using descriptive statistics. Cross-year differences in demographic variables were analyzed with Chi-square tests. We used Pearson correlations to look at associations between MRNI-A-r (EDD, AF, T), NTSM, and HCA scores. We used multivariable linear regression models to explore the linear trend in MRNI-A-r (EDD, AF, T) and NTSM scores over time. We also explored moderation of this change by Ethnocultural group. Finally, we used multivariable logistic regression models to explore homophobic name-calling by agent at different time points. Diagnostic checks were performed for model validation. Analyses were conducted in SPSS V29 and RStudio 2023.06.0 + 421.

## 3 Results

### 3.1 Sample descriptives

Summaries of demographic characteristics overall and by year are presented in Table 1. All participants in this project were in the 9th grade, with a mean age (SD) of 14.39 (0.37) (Table 1). The sample was quite racially/ethnically diverse, with 61.3% of the sample reporting that they were from a White population group only (Table 1). There were some differences in Ethnocultural group across years. Specifically, significantly fewer youth reported a multi-ethnic identity in fall 2013 and fall 2022; significantly more reported a multi-ethnic identity in fall 2016; and significantly fewer reported a racialized identity in fall 2021 [*X*^2^ (16, *N* = 855) = 46.82, *p* < 0.001]. Approximately two-thirds of participants lived in a dual caregiver home, and 57.1% had ever dated (Table 1). There was also a significant difference in dating across years, with participants in fall 2017 significantly more likely to report dating experience than in other years [*X*^2^ (8, *N* = 867) = 26.64, *p* < 0.001].

**Table 1:** Sample demographics for cisgender, heterosexual boys from 2013 to 2022.

	Full Sample (*N* = 873)	Fall 2013 (*n* = 37)	Fall 2014 (*n* = 117)	Fall 2015 (*n* = 142)	Fall 2016 (*n* = 111)	Fall 2017 (*n* = 128)	Fall 2018 (*n* = 15)	Fall 2019 (*n* = 156)	Fall 2021 (*n* = 78)	Fall 2022 (*n* = 89)
Age (years), mean (SD)	14.39 (0.37)	14.47 (0.41)	14.33 (0.33)	14.44 (0.44)	14.37 (0.38)	14.35 (0.34)	14.53 (0.33)	14.37 (0.32)	14.48 (0.41)	14.37 (0.35)
Population group, % (*n*)^a^										
African^b^	2.6 (22)	2.7 (1)	0.9 (1)	4.2 (6)	5.1 (5)	0.8 (1)	6.7 (1)	3.2 (5)	0.0 (0)	2.2 (2)
East Asian^b^	2.9 (25)	8.1 (3)	2.6 (3)	2.8 (4)	1.0 (1)	4.1 (5)	0.0 (0)	0.0 (0)	6.4 (5)	4.5 (4)
Filipino^b^	0.7 (6)	0.0 (0)	0.0 (0)	0.0 (0)	1.0 (1)	0.0 (0)	0.0 (0)	1.3 (2)	1.3 (1)	2.2 (2)
Indigenous^b^	4.8 (41)	–^c^	–^c^	–^c^	–^c^	–^c^	–^c^	–^c^	–^c^	–^c^
Latin American^b^	2.0 (17)	0.0 (0)	3.4 (4)	2.8 (4)	1.0 (1)	6.7 (1)	6.7 (1)	1.3 (2)	0.0 (0)	3.4 (3)
West Asian^b^	3.4 (29)	0.0 (0)	8.5 (10)	4.9 (7)	3.1 (3)	4.1 (5)	0.0 (0)	1.9 (3)	1.3 (1)	0.0 (0)
South Asian^b^	6.2 (53)	0.0 (0)	9.4 (11)	11.3 (16)	4.1 (4)	7.3 (9)	0.0 (0)	3.2 (5)	1.3 (1)	7.9 (7)
Southeast Asian^b^	1.8 (15)	2.7 (1)	5.1 (6)	2.8 (4)	1.0 (1)	0.8 (1)	0.0 (0)	0.0 (0)	0.0 (0)	2.2 (2)
White	61.3 (524)	73.0 (27)	58.1 (68)	50.7 (72)	53.1 (52)	55.3 (68)	60.0 (9)	64.7 (101)	80.8 (63)	71.9 (64)
Other^b^	3.7 (32)	10.8 (4)	2.6 (3)	0.7 (1)	8.2 (8)	4.9 (6)	13.3 (2)	4.5 (7)	0.0 (0)	1.1 (1)
Multi-ethnic^b^	10.6 (91)	0.0 (0)	6.0 (7)	14.1 (20)	17.3 (17)	13.8 (17)	6.7 (1)	12.8 (20)	7.7 (6)	3.4 (3)
Family structure, % (*n*)										
Dual caregiver	70.4 (601)	64.9 (24)	70.1 (82)	75.4 (107)	66.7 (74)	61.2 (71)	93.3 (14)	71.9 (110)	71.8 (56)	74.1 (63)
Other	29.6 (253)	35.1 (13)	29.9 (35)	24.6 (35)	33.3 (37)	38.8 (45)	6.7 (1)	28.1 (43)	28.2 (22)	25.9 (22)
Ever dated, % (*n*)^d^										
Yes	57.1 (495)	56.8 (21)	59.8 (70)	53.5 (76)	59.5 (66)	74.6 (94)	60.0 (9)	53.8 (84)	49.3 (37)	43.8 (38)
No	42.9 (372)	43.2 (16)	40.2 (47)	46.5 (66)	40.5 (45)	25.4 (32)	40.0 (6)	46.2 (72)	50.7 (38)	56.2 (50)
WiseGuyz participant, % (*n*)										
Yes	81.8 (714)	100.0 (37)	100.0 (117)	100.0 (142)	100.0 (111)	100.0 (128)	100.0 (15)	51.9 (81)	50.0 (39)	49.4 (44)
No	18.2 (159)	0.0 (0)	0.0 (0)	0.0 (0)	0.0 (0)	0.0 (0)	0.0 (0)	48.1 (75)	50.0 (39)	50.6 (45)
Population center type, % (*n*)										
Large	64.6 (564)	100.0 (37)	100.0 (117)	100.0 (142)	100.0 (111)	100.0 (128)	0.0 (0)	0.0 (0)	26.9 (21)	9.0 (8)
Medium^e^	18.6 (162)	0.0 (0)	0.0 (0)	0.0 (0)	0.0 (0)	0.0 (0)	100.0 (15)	59.0 (92)	37.2 (29)	29.2 (26)
Small^e^	16.8 (147)	0.0 (0)	0.0 (0)	0.0 (0)	0.0 (0)	0.0 (0)	0.0 (0)	41.0 (64)	35.9 (28)	61.8 (55)

^a^Significant cross year difference, *p* < 0.001. There were (a) significantly fewer multi-ethnic participants in 2013 and 2022; (b) significantly more multi-ethnic participants in 2016; (c) significantly fewer racialized participants in 2021; and (d) significantly more White participants in 2021 than would be expected by chance. By racialized youth, we mean youth who did not report White or Multi-ethnic as their population group.^b^Combined into one group (Ethnocultural youth) for multivariable analysis. ^c^Per Canadian ethics guidelines, we do not report per year sample size for Indigenous participants. ^d^Significant cross year difference, *p* < 0.001. There were significantly fewer non-daters in 2017 than would be expected by chance. ^e^Combined into one group (Small/Medium) for multivariable analysis.

When exploring if NTSM, MRNI-A-r, or HCA scores differed by (1) WiseGuyz participation status or (2) the type of population center where the participant attended school, we did not find any differences by WiseGuyz participation status (Supplementary Table S2). However, we found that toughness scores (MRNI-A-r: T), homophobic attitudes (NTSM), and total use of homophobic name-calling (HCA) were all higher for participants who attended schools in small/medium population centers, as compared to large population centers (Supplementary Table S2). For NTSM and HCA scores, this difference was driven by participants residing in small population centers specifically (Supplementary Table S2). Thus, in analyses reported below, we controlled for population center type, as well as dating status and Ethnocultural group, as relevant.

### 3.2 Overt homophobic attitudes and adherence to related patriarchal norms by year

**Figure 1:**
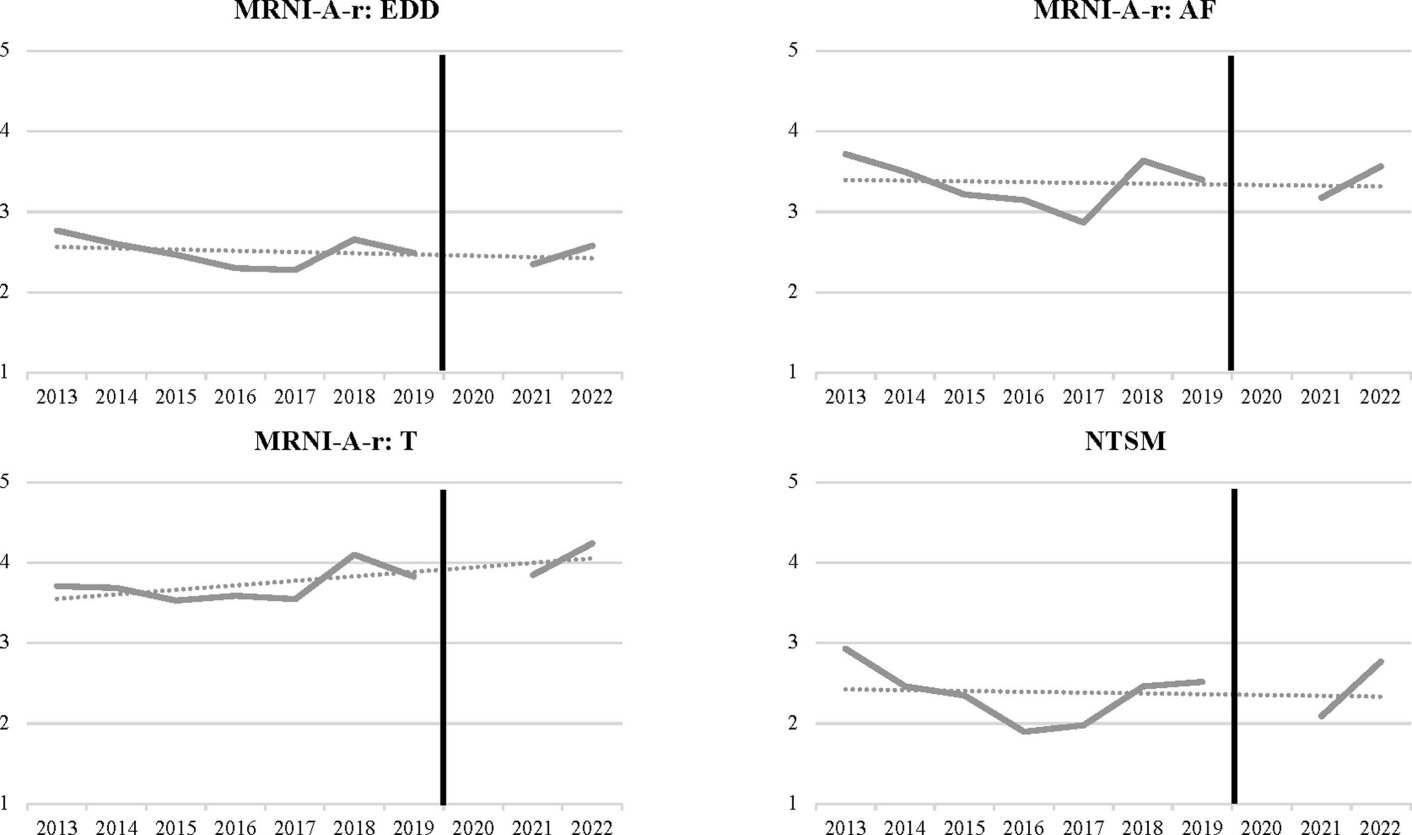
Visualization of MRNI-A-R EDD, AF, T and NTSM average scores over time. For all panels, measurement is on a 7-point scale were 1-strongly disagree and 7-strongly agree. We use average scale scores instead of sum scores in this figure for each of interpretation and comparison. Solid line represents average scores in the respective scale for the given year. There is a gap in 2020 as no data were collected due to COVID-related school closures. Dotted line visualizes linear trend. Data to left of solid black line on each graph is what is modeled in Tables 2, 3 (2013–2019). AF, Avoidance of femininity; EDD, Emotionally detached dominance; T, Toughness; NTSM, Negativity toward sexual minorities.

Mean scores by year for overt homophobic attitudes (NTSM) and adherence to related patriarchal norms (MRNI-A-r: EDD, MRNI-A-r: AF, MRNI-A-r: T) are visualized in Figure 1. To explore differences in NTSM and MRNI-A-r scores across time, we used two approaches. First, we examined if there was change over time in any of these scores from 2013 to 2019, using multivariable linear regression models. Results from these models are presented in Tables 2, 3. We found significant declines from 2013–2019 in overt homophobic attitudes (NTSM), as well as in emotionally detached dominance (MRNI-A-r: EDD) and avoidance of femininity (MRNI-A-r: AF) scores. However, we did not find a decline in toughness scores (MRNI-A-r: T) across this period. We also found that boys who attended schools in small/medium populations centers reported higher NTSM, MRNI-A-r: EDD, MRNI-A-r: AF, and MRNI-A-r: T scores, as compared to boys attending school in a large population center, and that boys who had dated reported higher MRNI-A-r: EDD scores as compared to boys who had not dated or were not sure (Tables 2, 3).

**Table 2:** Multivariable linear regression models exploring NTSM scale scores over time, 2013–2019.

	NTSM		
	b (SE)	95% CI	*p*-value
Intercept	**23.61 (1.51)**	**20.65, 26.57**	**<0.001**
Time	**−1.70 (0.40)**	**−2.49, −0.91**	**<0.001**
Small/Medium Population Center	**8.29 (1.78)**	**4.79, 11.79**	**<0.001**
Ethnocultural (yes)	**2.05 (0.89)**	**0.31, 3.80**	**0.021**
Dater (yes)	−1.63 (0.90)	−3.40, 0.13	0.069

For population center type, comparison group is Large. For Ethnocultural group, comparison group is White youth. SE, Standard error; CI, Confidence interval; NTSM, Negativity toward sexual minorities. Bold values indicate statistically significant findings.

**Table 3:** Multivariable linear regression models exploring MRNI-A-r sub-scale scores over time, 2013–2019.

	EDD			AF			T		
	b (SE)	95% CI	*p*-value	b (SE)	95% CI	*p*-value	b (SE)	95% CI	*p*-value
Intercept	**43.38 (2.02)**	**39.42, 47.34**	**<0.001**	**22.76 (1.11)**	**20.59, 24.93**	**<0.001**	**25.39 (1.08)**	**23.27, 27.51**	**<0.001**
Time	**−2.26 (0.54)**	**−3.31, −1.20**	**<0.001**	**−1.36 (0.29)**	**−1.94, −0.78**	**<0.001**	−0.42 (0.29)	−0.98, 0.15	0.15
Small/Medium Population Center	**9.49 (2.40)**	**4.78, 14.20**	**<0.001**	**6.14 (1.31)**	**3.57, 8.72**	**<0.001**	**3.31 (1.28)**	**0.79, 5.83**	**0.010**
Ethnocultural (yes)	**3.47 (1.19)**	**1.13, 5.81**	**0.0037**	0.73 (0.65)	−0.55, 2.02	0.26	0.90 (0.64)	−0.36, 2.15	0.16
Dater (yes)	**2.35 (1.20)**	**0.084, 4.81**	**0.042**	1.07 (0.66)	−0.23, 2.36	0.11	1.24 (0.65)	−0.029, 2.51	0.056

For population center type, comparison group is Large. For Ethnocultural group, comparison group is White youth. SE, Standard error; CI, Confidence interval; AF, Avoidance of femininity; EDD, Emotionally detached dominance; T, Toughness. Bold values indicate statistically significant findings.

As we were unable to collect data in fall 2020 due to COVID-19, we could not explore an uninterrupted linear trend from 2013 to 2022. Instead, to determine if scores in 2021 and 2022 were significantly different than scores in 2013, we used multivariable linear regression models with a year-wise comparison (Tables 4, 5). In these models, we found that NTSM scores were significantly lower in fall 2021 than in fall 2013 (and somewhat lower in fall 2022; Table 4), suggesting the declining linear trend in overt homophobic attitudes may have continued in the post-COVID period. Conversely, MRNI-A-r: EDD and MRNI-A-r: AF scores were not significantly lower in 2021 or 2022 than in fall 2013, suggesting the declining linear trend we found from 2013–2019 may not have continued post-COVID (Table 5). For MRNI-A-r: T scores, we found that these scores were significantly higher in fall 2022 than in fall 2013, suggesting a potential increase in the post-COVID period (Table 5).

**Table 4:** Multivariable linear regression model exploring NTSM scale scores at distinct time points (year-wise comparison), 2013–2022.

	NTSM		
	b (SE)	95% CI	*p*-value
Intercept	**23.40 (1.91)**	**19.64, 27.15**	**<0.001**
Fall 2014	−4.00 (2.12)	−8.16, 0.16	0.059
Fall 2015	**−5.08 (2.08)**	**−9.16, −1.01**	**0.015**
Fall 2016	**−8.58 (2.17)**	**−12.85, −4.31**	**<0.001**
Fall 2017	**−7.90 (2.12)**	**−12.06, −3.74**	**0.00021**
Fall 2018	−7.87 (4.19)	−16.08, 0.35	0.060
Fall 2019	**−7.47 (3.15)**	**−13.66, 1.28**	**0.018**
Fall 2020	–	–	–
Fall 2021	**−9.45 (2.83)**	**−14.99, −3.90**	**0.00087**
Fall 2022	−5.15 (3.09)	−11.22, 0.92	0.096
Small/Medium Pop. Center	3.89 (2.39)	−0.80, 8.58	0.10
Ethnocultural (yes)	**2.07 (0.81)**	**0.47, 3.67**	**0.011**
Dater (yes)	−0.97 (0.80)	−2.54, 0.59	0.22

Comparison year was fall 2013. Data were not collected in fall 2020 due to COVID-19. We include a placeholder for this year in this table to make this gap clear. For population center type, comparison group is Large. For Ethnocultural group, comparison group is White youth. SE, Standard error; CI, Confidence interval; NTSM, Negativity toward sexual minorities. Bold values indicate statistically significant findings.

**Table 5:** Multivariable linear regression model exploring MRNI-A-r sub-scale scores at distinct time points (year-wise comparison), 2013–2022.

	EDD			AF			T		
	b (SE)	95% CI	*p*-value	b (SE)	95% CI	*p*-value	b (SE)	95% CI	*p*-value
Intercept	**41.82 (2.55)**	**36.82, 46.82**	**<0.001**	**21.41 (1.43)**	**18.59, 24.22**	**<0.001**	**24.93 (1.38)**	**22.22, 27.63**	**<0.001**
Fall 2014	−3.27 (2.82)	−8.80, 2.27	0.25	−1.40 (1.59)	−4.52, 1.71	0.38	−0.27 (1.53)	−3.27, 2.72	0.86
Fall 2015	−5.40 (2.77)	−10.83, 0.031	0.051	**−3.06 (1.56)**	**−6.12, −0.007**	**0.049**	−1.37 (1.50)	−4.31, 1.56	0.36
Fall 2016	**−8.02 (2.90)**	**−13.71, −2.32**	**0.0059**	**−3.46 (1.63)**	**−6.67, −0.26**	**0.034**	−0.60 (1.57)	−3.68, 2.48	0.70
Fall 2017	**−9.57 (2.83)**	**−15.12, −4.02**	**0.00075**	**−5.78 (1.59)**	**−8.90, −2.66**	**0.00030**	−1.81 (1.53)	−4.81, 1.19	0.24
Fall 2018	1.18 (5.66)	−9.93, 12.27	0.84	−1.19 (3.18)	−7.44, 5.05	0.71	4.60 (3.01)	−1.84, 7.05	0.14
Fall 2019	−1.20 (4.19)	−9.42, 7.02	0.77	−2.30 (2.35)	−6.92, 2.32	0.33	2.61 (2.26)	−1.84, 7.05	0.26
Fall 2020	–	–	–	–	–	–	–	–	–
Fall 2021	−3.99 (3.76)	−11.38, 3.39	0.29	−3.40 (2.12)	−7.56, 0.75	0.11	2.57 (2.04)	−1.43, 6.57	0.21
Fall 2022	0.51 (4.11)	−7.55, 8.57	0.90	−1.08 (2.31)	−5.61, 3.45	0.64	**5.48 (2.22)**	**1.12, 9.84**	**0.014**
Small/Medium Pop. Center	−3.70 (3.16)	−9.91, 2.50	0.24	0.25 (1.78)	−3.24, 3.73	0.89	−1.97 (1.71)	−5.33, 1.39	0.25
Ethnocultural (yes)	**3.10 (1.08)**	**0.97, 5.23**	**0.0043**	0.59 (0.61)	−0.61, 1.78	0.33	0.66 (0.59)	−0.50, 1.81	0.26
Dater (yes)	**2.93 (1.07)**	**0.84, 5.03**	**0.0060**	**1.29 (0.60)**	**0.11, 2.46**	**0.032**	**1.53 (0.58)**	**0.40, 2.66**	**0.00081**

Comparison year was fall 2013. Data were not collected in fall 2020 due to COVID-19. We include a placeholder for this year in this table to make this gap clear. For population center type, comparison group is Large. For Ethnocultural group, comparison group is White youth. SE, Standard Error; CI, Confidence Interval; AF, Avoidance of Femininity; EDD, Emotionally detached dominance; T, Toughness. Bold values indicate statistically significant findings.

For our planned moderation analysis by Ethnocultural group, we did find a main effect of Ethnocultural group on NTSM (Table 2) and MRNI-A-r: EDD (Table 3) scores from 2013–2019, indicating that Ethnocultural youth reported greater overall overt homophobic attitudes and adherence to norms supporting emotional restriction as compared to White boys in our sample. However, we did not find significant moderation between Ethnocultural group and time for either NTSM or MRNI-A-r: EDD, indicating that Ethnocultural youth had significantly higher scores at all time points from 2013–2019 on these two scales.

### 3.3 Overall use of homophobic name-calling and association with overt homophobic attitudes

When exploring correlations between the HCA total score, indicating the overall amount of homophobic name-calling the participant used (regardless of who this name-calling was aimed at), and NTSM scores, we found that the correlation was significant, but as hypothesized, small in magnitude (*r* = 0.33; Table 6). We also explored this correlation for White and Ethnocultural boys separately (Supplementary Table S3). Although the correlation between HCA (total) and NTSM scores was small and significant for both White and Ethnocultural boys, the magnitude of the correlation was smaller for Ethnocultural boys, indicating a weaker relationship (*r*, White boys = 0.39; *r*, Ethnocultural boys = 0.21; Supplementary Table S3).

**Table 6:** Correlations^a^.

	1.	2.	3.	4.	5.	6.	7.	8.	9.	10.
NTSM	**–**	0.52^***^	0.70^***^	0.40^***^	0.33^***^	0.22^***^	0.26^***^	0.23^***^	0.21^***^	0.25^***^
MRNI-A-r: EDD	0.57^***^	**–**	0.68^***^	0.71^***^	0.26^***^	0.21^***^	0.21^***^	0.21^***^	0.098^*^	0.11^*^
MRNI-A-r: AF	0.68^***^	0.72^***^	**–**	0.64^***^	0.33^***^	0.26^***^	0.24^***^	0.29^***^	0.13^**^	0.17^***^
MRNI-A-r: T	0.45^***^	0.73^***^	0.68^***^	**–**	0.29^***^	0.26^***^	0.15^***^	0.28^***^	0.078	0.13^**^
HCA total score	0.33^***^	0.26^***^	0.33^***^	0.29^***^	**–**	0.76^***^	0.70^***^	0.74^***^	0.63^***^	0.57^***^
HCA: Friend	0.22^***^	0.21^***^	0.26^***^	0.26^***^	0.76^***^	**–**	0.29^***^	0.48^***^	0.25^***^	0.19^***^
HCA: Do not like	0.26^***^	0.21^***^	0.24^***^	0.15^***^	0.70^***^	0.29^***^	–	0.35^***^	0.51^***^	0.43^***^
HCA: Not gay	0.23^***^	0.21^***^	0.29^***^	0.29^***^	0.74^***^	0.48^***^	0.35^***^	–	0.28^***^	0.34^***^
HCA: Do not know	0.21^***^	0.098^*^	0.13^**^	0.078	0.63^***^	0.25^***^	0.51^***^	0.28^***^	–	0.36^***^
HCA: Thought gay	0.25^***^	0.11^*^	0.17^***^	0.13^**^	0.57^***^	0.19^***^	0.43^***^	0.34^***^	0.36^***^	–

***p < 0.001; **p < 0.01; *p < 0.05. AF, Avoidance of Femininity; EDD, Emotionally detached dominance; T, Toughness; NTSM, Negativity Toward Sexual Minorities; HCA, Homophobic Content Agent. Correlations >0.50 (i.e., a moderate correlation or larger) are bolded in the table to support interpretation. ^a^Below the diagonal: bivariate correlations for full sample. Above the diagonal: bivariate correlations for participants with data from 2016–2022 when HCA data were collected (*n* = 563).

### 3.4 Differential use of homophobic name-calling

Like with the HCA total score, we found that correlations between HCA Agent sub-scales and NTSM scores were significant but small in magnitude [*r* range: 0.21–0.26; Table 6]. The largest correlation with NTSM scores was for the HCA “do not like” sub-scale (*r* = 0.26), and the smallest was for the HCA “do not know” sub-scale (*r* = 0.21; Table 6). Correlations between HCA sub-scales were generally small in magnitude, as well (*r* range: 0.19–0.51; Table 6). The largest correlations were between HCA “do not know” sub-scale scores and HCA “do not like” sub-scale scores (*r* = 0.51), and between HCA “not gay” sub-scale scores and HCA “friend” sub-scale scores (*r* = 0.48). The smallest correlation was between HCA “thought gay” sub-scale scores and HCA “friend” sub-scale scores (*r* = 0.19).

**Figure 2:**
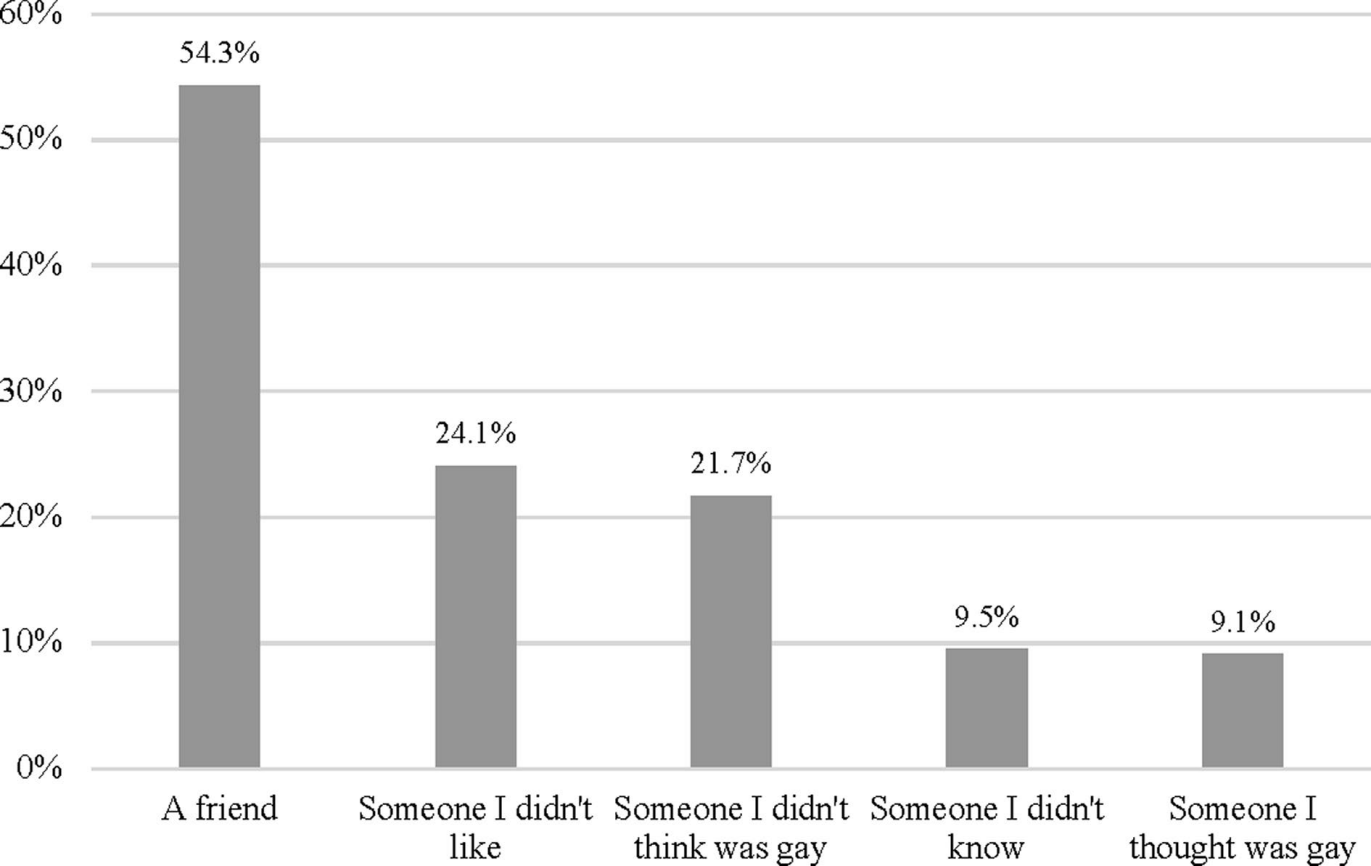
Percentage of cisgender, heterosexual participants who indicated any form of homophobic name-calling by agent, 2016–2022 (*n* = 562).

Finally, analyzing agents toward which homophobic name-calling was used, we found that participants across all years most commonly used homophobic name-calling toward a friend (54.3%), followed by someone they did not like (24.1%) someone they did not think was gay (21.7%), someone they did not know (9.5%), and someone they thought was gay (9.1%). This suggests that, as expected, homophobic name-calling was used in a differential manner by participants in this sample (Figure 2). In supplementary analyses, we explored whether there were any differences in homophobic name-calling by Ethnocultural group, or the type of population center where the participant attended school. There were no differences in HCA sub-scale scores by Ethnocultural group, and the overall pattern of name-calling was overall the same as for the sample as a whole, with friends being by far the most common target (Supplementary Figure S1). For population center, we found that boys who attended schools in small population centers were more likely to use homophobic name-calling toward a friend or someone they thought was gay, and that boys who attended schools in large population centers were less likely to use homophobic name-calling toward someone they did not think was gay. However, as for Ethnocultural boys, the overall pattern of name-calling was the same as in the overall sample (Supplementary Figure S2). There were also no cross-year differences in name-calling by Agent, in models controlling for Ethnocultural group, population center size, and dating status (Table 7).

**Table 7:** Multivariable logistic regression model exploring HCA sub-scale scores at distinct time points (year-wise comparison), 2016–2022.

Agent	Friend			Someone I did not like			Someone I did not think was gay		
	aOR	95% CI	*p*-value	aOR	95% CI	*p*-value	aOR	95% CI	*p*-value
Intercept	**0.48**	**0.25, 0.90**	**0.024**	**0.30**	**0.14, 0.60**	**0.00083**	**0.15**	**0.059, 0.32**	**<0.001**
Fall 2017	0.85	0.44, 1.66	0.64	0.57	0.26, 1.24	0.15	0.83	0.34, 2.17	0.69
Fall 2018	–	–	–	–	–	–	–	–	–
Fall 2019	1.31	0.46, 3.83	0.62	0.72	0.22, 2.34	0.59	1.02	0.28, 3.69	0.97
Fall 2020	–	–	–	–	–	–	–	–	–
Fall 2021	1.59	0.62, 4.15	0.34	0.88	0.29, 2.52	0.81	2.14	0.66, 6.85	0.20
Fall 2022	2.00	0.70, 5.75	0.20	1.04	0.31, 3.28	0.94	1.22	0.34, 4.35	0.76
S/M Pop. Center	1.37	0.58, 3.12	0.47	1.30	0.52, 3.59	0.60	1.69	0.67, 4.72	0.29
Ethnocultural (yes)	1.21	0.82, 1.80	0.33	1.10	0.70, 1.70	0.68	1.10	0.69, 1.76	0.68
Dater (yes)	**2.08**	**1.42, 3.07**	**0.00020**	1.25	0.81, 1.93	0.32	1.26	0.81, 1.99	0.31
Agent	Someone I did not know				Someone I thought was gay				
	aOR	95% CI		*p*-value	b (SE)		95% CI		*p*-value
Intercept	**0.067**	**0.018, 0.19**		**<0.001**	**0.025**		**0.0038, 0.093**		**<0.001**
Fall 2017	0.74	0.21, 2.96		0.65	1.22		0.27, 8.57		0.81
Fall 2018	–	–		–	–		–		–
Fall 2019	1.05	0.16, 6.16		0.96	0.63		0.026, 7.71		0.72
Fall 2020	–	–		–	–		–		–
Fall 2021	1.20	0.21, 6.12		0.83	0.92		0.041, 10.19		0.95
Fall 2022	2.29	0.38, 12.52		0.34	1.21		0.052, 14.47		0.88
S/M Pop. Center	1.21	0.35, 5.58		0.78	4.87		0.91, 90.55		0.14
Ethnocultural (yes)	1.33	0.69, 2.50		0.38	1.09		0.55, 2.11		0.79
Dater (yes)	1.09	0.59, 2.06		0.78	1.83		0.96, 3.60		0.073

Comparison year was fall 2016. Data were not collected in fall 2020 due to COVID-19. We include a placeholder for this year in this table to make this gap clear. Data on this scale were also not collected on the fall 2018 survey, but we include a placeholder for this year in this table to make this gap clear. For population center type, comparison group is Large. For Ethnocultural group, comparison group is White youth. S/M, Small/Medium; aOR, Adjusted Odds Ratio; CI, Confidence interval. Bold values indicate statistically significant findings.

## 4 Discussion

This study provides a quantitative exploration of the ideas of hybrid masculinities in a North American sample. We hypothesized that if the ideas of hybrid masculinities applied in our sample, we would find that overt homophobic attitudes and adherence to related patriarchal norms would decline over time, but that homophobic name-calling – the actual practice used to regulate masculinities in Western settings – would not be strongly correlated with overt homophobic attitudes, and that because of its role in policing masculinity among peers, would be differentiated in its use (Pascoe, 2013). We also explored how findings differed for a specific sub-group – Ethnocultural boys – to see if there were differences that might be related to perceptions of marginalized masculinities. Our findings generally supported our hypothesis, though we did not find many differences between White and Ethnocultural boys in our sample.

Our first hypothesis regarding the decline in overt homophobic attitudes and related patriarchal norms was mostly supported. Specifically, we found that overt homophobic attitudes declined significantly from 2013 to 2019, and that this decline appeared to continue in the post-COVID period. This decline also reflects larger trends in North America (Flores, 2014; Pew Research Center, 2019). It is important to note that in our sample, even in 2013, overall levels of overt homophobic attitudes were quite low, with a mean scale score around ‘somewhat disagree’. In part, this may be because the NTSM is a very explicit measure, which – because of changing societal attitudes more broadly – may lead to issues with social desirability in responding. Because of this lower overall mean score, we were likely somewhat limited in our ability to detect change. To this end, in future work, we recommend also using more implicit measures of homophobia/sexual prejudice to assess homophobic attitudes among participants (e.g., Poteat et al., 2015). Despite this limitation, we still saw a significant decline over the 10-year study period, with a mean score closer to ‘disagree’ by fall 2022.

We also found a linear decline in adherence to norms supporting emotional restriction and avoidance of femininity from 2013–2019. We chose to explore these norms in addition to overt homophobic attitudes since research on masculinities has repeatedly pointed to the interactional relationship between masculinity and homophobia (Phoenix et al., 2003; Pascoe, 2007; Bridges and Pascoe, 2014; Diefendorf and Bridges, 2020). Overall, then, it is not surprising that these scores showed similar declines to homophobic attitudes. However, we did not find a change in adherence in toughness norms from 2013–2019. The ‘toughness’ sub-scale also consistently had the highest average score of all scales over the study period (average scale score around ‘somewhat agree’). In terms of why we did not find a decline in adherence to toughness norms, it is possible this scale does not cover homophobic-adjacent concepts in the same way that the emotional restriction and avoidance of femininity scales do. For example, in the original measure validation study with early adolescents (Levant et al., 2012), five of the seven items on the ‘toughness’ scale came from items originally designed to tap aggression, self-reliance, and achievement/status, whereas only one came from items designed to tap restrictive emotionality and one from items designed to tap avoidance of femininity. Comparatively, eight of the 16 items on the ‘emotionally detached dominance’ scale came from items designed to tap restrictive emotionality/avoidance of femininity, and all six items on the ‘avoidance of femininity’ scale came from items designed to tap avoidance of femininity. It is also possible that the ‘toughness’ items, which primarily focus on defending oneself, trying to be the best, and gaining respect/admiration, are still more socially acceptable than avoidance of femininity or emotional restriction items, and so were more resistant to change.

Data also suggest some changes in the post-COVID-19 period. Specifically, data from 2021 to 2022 suggest that the linear decline in adherence to emotional restriction and avoidance of femininity norms may not have continued in the post-COVID period, and that there was an increase in adherence to toughness norms. The lack of continued decline might reflect the increasing influence of the manosphere during and post-COVID. As noted by Barker et al. (2021), due to the increased amount of time men and boys spent online during the pandemic, “the politics of online angry manhood and antifeminist sentiment may have increased during COVID-19…social isolation and the deliberate right-wing politicization of some men’s increasing economic precarity suggest 2020 was a particularly fertile year for [manosphere] expansion efforts” (p. 171). Research from South Asia also demonstrates a significant increase in the percentage of misogynistic tweets since 2020 (Dehingia et al., 2021). Anecdotally, WiseGuyz program facilitators also have reported on the rise in popularity of figures like Andrew Tate since the pandemic, and a recalcitration of patriarchal attitudes among program participants that seemed to be declining pre-pandemic.

Our second hypothesis, that the correlation between overt homophobic attitudes and homophobic name-calling (overall and by agent) would be small in magnitude, and that homophobic name-calling by agent would be differentiated, was supported. Specifically, although overt homophobic attitudes declined, homophobic name-calling remained differentiated, with significantly higher name-calling toward a friend as compared to someone youth thought was gay. One limitation of these data is that we cannot know *why* youth engaged in this homophobic name-calling. Currently in the literature, we note two possible interpretations for this use. One possible interpretation is that of Anderson and McCormack (McCormack et al., 2016; Anderson and McCormack, 2018), which posits that some youth may use homosexually-themed language toward friends as a demonstration of friendship closeness and playfulness. Yet, as feminist scholars, we feel that even if used to be ‘playful’, this type of discourse nonetheless exposes youth (including the agent, target and bystanders) to language that reinforces patriarchal masculine norms and fortifies what are appropriate ways to be (and not be) a boy (Pascoe, 2007; Reigeluth and Addis, 2015; Bridges and Pascoe, 2018). The second interpretation (and the one explored in this paper) is from Pascoe (2013) and Bridges and Pascoe (2014, 2016, 2018), and agrees with Anderson and McCormack that homophobic name-calling is not about overt homophobia. However, Bridges and Pascoe differ in their discussion of why this name-calling is used toward a friend, specifically stating that friend-targeted use is a way of using jokes, taunts and imitations to punish those who transgress gender norms, and not the result of innocuous social use (Pascoe, 2013). Thus, in this interpretation, homophobic name-calling has as much to do with failing to appear competent in stereotypical masculine behaviors (e.g., heterosexual prowess) as it does with sexual identity (Pascoe, 2013). Indeed, we feel that a core issue with the idea that homophobic discourse is used for social bonding is that it is still using a less dominant group to ‘other’ peers.

Based on our data, and congruent with a significant body of research examining the intersections between homophobic harassment and adolescent masculinity (Phoenix et al., 2003; Pascoe, 2007; Carrera-Fernández et al., 2018; Munsch and Gruys, 2018), we contend that homophobic name-calling continues to act as a discursive strategy to police and discipline male gender practices and identities that are countertyped against culturally-valued masculinities (Diefendorf and Bridges, 2020). For example, the smallest correlation between HCA Agent-specific scores in our data was between name-calling toward someone the person thought was gay and name-calling toward a friend, suggesting these are distinct behaviors with different underlying motivations. Further, qualitative data collected from the 2014–2015 offering of WiseGuyz highlights a common realization among participants (post-program) about how the use of this language contributes to the perpetuation of patriarchal masculine norms (Hurlock, 2016). For example, one participant stated “I do not think they mean to be homophobic but ‘gay’ it’s just a word like ‘gay’ is a slur that everyone uses for some reasons to bring people down. Saying that like makes people that are gay, makes them not wanna come out and tell other people in case they are bullied” (Hurlock, 2016, p. 43).

Finally, exploring a sub-group identified as important in prior theory and research (Ethnocultural youth), we did find that Ethnocultural youth in our sample reported significantly more emotional restriction and overt homophobic attitudes, as compared to their White peers. This increased adherence to emotional restriction and homophobia may reflect an outcome of marginalized masculinities, given previous research pointing to hypermasculinity as a potential coping mechanism for racialized adolescents (e.g., O’Donnell and Sharpe, 2000; Spencer et al., 2004; Griffith et al., 2012). However, there was no difference between Ethnocultural and White youth in terms of homophobic name-calling (either overall or by agent), or in terms of adherence to the patriarchal norms of avoidance of femininity and toughness. There was also no interaction between the decline over time in homophobic attitudes or emotional restriction and Ethnocultural group. Thus, it appears that, although they reported higher average levels of overt homophobic attitudes and emotional restriction, Ethnocultural youth experienced a similar decline in these attitudes over time as White youth in our sample. In addition, both White and Ethnocultural youth in our sample used homophobic name-calling in a differentiated way. However, more research on the potential application of the ideas of hybrid masculinities to racialized youth in non-American contexts is needed to better understand these findings.

### 4.1 Theoretical implications

Overall, our data lend support to the framework of hybrid masculinities, suggesting that while there are positive shifts in relation to broader homophobic attitudes in this sample, homophobic name-calling among peers is likely still being deployed by some boys to emasculate and regulate other young men (Pascoe, 2013). Our data also support the process of fortifying boundaries, given that after friends, the next most common agent that homophobic name-calling was directed toward was someone the youth did not like. This action suggests that, for some boys, homophobic name-calling remains a way of identifying outsiders in the group, establishing social boundaries, and upholding unequal power relations (Pascoe, 2013; Bridges and Pascoe, 2018). In this way, homophobic name-calling can be used both as a strategy of repudiation and confirmation, rejecting a feminized identity as well as enforcing dominance over less powerful peers (Bridges and Pascoe, 2016). This finding also corresponds to arguments by critical masculinity scholars who point out that while homophobia and homophobic rhetoric is often publicly penalized and generally less socially acceptable than in past decades (at least in many Western contexts), there are still a range of micro-processes and interactions that continue to uphold, and reproduce, heterosexism and homophobia within different settings, such as schools (Eisen and Yamashita, 2019; Christofidou, 2021).

Our data are also the first (to our knowledge) to quantitatively explore the ideas of hybrid masculinities in a mid-adolescent sample. Understanding practices of masculinity at this critical juncture for the development of gender-based identities and behaviors is needed in the literature (Lomas et al., 2013, 2020). For example, a recent systematic review of perceptions and interpretations of contemporary masculinity found that none of the included articles had a sample younger than age 16 (Connor et al., 2021). Our sample of mid-adolescent boys (mean age 14.39) thus provides an important addition to the literature on contemporary practices of masculinity. Future research should continue to explore the ideas of hybrid masculinities with early, mid, and late adolescents, as well as how these ideas might intersect with other theories of contemporary practices (e.g., Inclusive Masculinity Theory; McCormack et al., 2016). This research should also focus on careful consideration of diverse groups of boys and the role their social location may play in their gendered practices, as there are likely a number of important nuances within the broad ideas of discursive distancing, strategic borrowing, and fortifying boundaries across intersections of boys’ identities (e.g., for bisexual boys; Winer, 2022).

Finally, we note that these data were not designed to be and are not a conclusive test of the ideas of hybrid masculinities. For example, as described above, in Inclusive Masculinity Theory, Anderson and McCormack state that homosexually-themed language is mostly used as social bonding, and not as masculinity policing (McCormack and Anderson, 2010; McCormack, 2011; McCormack et al., 2016; Anderson and McCormack, 2018). It is certainly possible to read our HCA data as capturing homosexually-themed language, and not targeted name-calling. However, we feel that if Inclusive Masculinity Theory was a better fit for our data, in addition to the overall decline in homophobic attitudes and small correlation between HCA “friend” and HCA “thought gay” scores that we found, we would also have found strong correlations between overt homophobic attitudes and name-calling toward someone the youth thought was gay, which we did not. Further, all HCA inter-correlations were small, suggesting participants are using this name-calling in different ways with different people. This suggests that, depending on context, the ideas of both hybrid masculinities and Inclusive Masculinity Theory might apply (e.g., some boys may be using this name-calling to regulate masculinity, others to bond, and still others for another purpose). A key empirical question for future research is thus to explore differences in motivation for homophobic name-calling across settings and contexts.

### 4.2 Limitations

A primary limitation is the nature of our sample. Specifically, although we collected data in the same metropolitan area from 2013 to 2022, we were not at the same schools in each year, and thus school differences could be contributing to the declines we see. In addition, as there was some overlap between the schools were WiseGuyz was offered across cohorts in this study, it is also possible that the continued presence of WiseGuyz in these institutional settings may be linked to some of the decline in homophobic attitudes over time. We also expanded data collection to include small and medium population centers starting in 2018, which could be driving some differences over time. However, to account for this change, we controlled for population center type in all multivariable models. We also only collected data in one fairly conservative province, and thus it is possible findings would be different in more politically liberal settings. In terms of our sample, we included boys who self-reported that they were cisgender and heterosexual, but we acknowledge that despite assurances of anonymity and confidentiality, some boys may have chosen to mask their true sexual and/or gender identity in their survey responses. A second limitation is that we only asked about homophobic name-calling starting in fall 2016, which limited our sample size for these analyses. Due to COVID-related school closures, we were unable to collect data in fall 2020, so could not look at an uninterrupted linear trend from 2013–2022. All data were also self-report, and thus subject to social desirability bias. We were only able to explore effects for Ethnocultural and White youth, as we did not have the power to explore effects by individual Ethnocultural groups in this sample. Thus, it is important to note that our results should not be interpreted as compared to a White ‘norm’. Intersectional distinctions are important considerations in relation to inequitable access to forms of hybrid masculinities (Bridges and Pascoe, 2014). Our inability to look at distinct Ethnocultural groups may also be the reason for the limited findings pertaining to this sub-sample. Finally, we looked at the broad ideas of hybrid masculinities, but note that these ideas are not binary, and that there is likely important context and nuance within each of these ideas (e.g., some young men may borrow aspects of feminized masculinities to obtain hegemonic goals like sexual conquest in some settings, and to experience more intimate relationships with people of all genders in others). Understanding this continuum of engagement with hybrid masculinities is an important area for future research.

## 5 Conclusion

Hegemonic masculinities are relational and discursive, and for these reasons, they are subject to change both within an individual person’s life course and over time (Connell and Messerschmidt, 2005). Although there appears to be a widening range of practices and performances of contemporary masculinities, such that on the surface many boys and men seem to be moving away from rigid and stereotypical forms, the ideas of hybrid masculinities would suggest that for many heterosexual, cisgender boys, the underlying hierarches and power relations remain. In this sample of heterosexual, cisgender mid-adolescent boys from one province in Western Canada, we did find that overt homophobic attitudes declined significantly over a 10-year period. Given the relationship between homophobia and masculinity, this decline could be viewed as a manifestation of meaningful shifts in how masculinities are experienced and expressed. However, as stated by Bridges and Pascoe, “privilege works best when it goes unrecognized” (2014, p. 256). Indeed, while overt negative attitudes regarding sexual minorities declined significantly in our sample, acts of homophobic name-calling persisted, and appeared to continue being used by some boys as a gender policing tool, fortifying boundaries and upholding unequal power relations. Hybrid masculinities, and the process of fortifying boundaries, appears well suited to account for these findings of both change and resistance.

## Data availability statement

The datasets presented in this article are not readily available because of data confidentiality requirements. Requests to access the datasets should be directed to deinera.exner2@ucalgary.ca.

## Ethics statement

The studies involving humans were approved by the University of Calgary Conjoint Faculties Research Ethics Board. The studies were conducted in accordance with the local legislation and institutional requirements. Written informed consent for participation in this study was provided by the participants’ legal guardians.

## Author contributions

DE-C: Conceptualization, Formal analysis, Methodology, Writing – original draft. CC: Conceptualization, Writing – review & editing. AJ: Conceptualization, Writing – review & editing. VV: Formal analysis, Writing – review & editing.
