# Supplementary material for: Homophobic beliefs and attitudes among mid-adolescent boys: exploring the ideas of hybrid masculinities
Source: Front Sociol. 2024 Jun 11;9:1347568. doi: 10.3389/fsoc.2024.1347568 (PMC11196794; doi:10.3389/fsoc.2024.1347568)
Supplement: Supplementary file 1 [file Data_Sheet_1.docx]

Supplementary Material

**Supplemental Table 1**

***Flowchart of Study Sample Across Study Years***

Year (Total Sample Size)

Analysis Sample Size (Cisgender, Heterosexual Boys)

Sample Size for Each Measure

^a^ Sample size is much smaller this year as we had a change in grant funding, and as a result only collected pilot data from one new school division in fall 2018.

^b^ In fall 2017, we only collected HCA data from a randomly selected third of the total sample. We did not collect HCA data in fall 2014, 2015, 2016, or 2018.

**Supplemental Table 2**

***Comparison of Scale Scores by WiseGuyz Participation and Population Center Type***

|  | MRNI-A-r: EDD | | | | MRNI-A-r: AF | | | | MRNI-A-r: T | | | | NTSM | | | | HCA | | | |
| --- | --- | --- | --- | --- | --- | --- | --- | --- | --- | --- | --- | --- | --- | --- | --- | --- | --- | --- | --- | --- |
|  | N | Mean (SD) | *p* | *\|d\|* | N | Mean (SD) | *p* | *\|d\|* | N | Mean (SD) | *p* | *\|d\|* | N | Mean (SD) | *p* | *\|d\|* | N | Mean (SD) | *p* | *\|d\|* |
| **WiseGuyz Participant** | | | | | | | | | | | | | | | | | | | | |
| Yes | 701 | 39.30 (15.54) | .73 | .031 | 704 | 19.66 (8.63) | .62 | .044 | 705 | 25.98 (8.41) | .15 | .091 | 705 | 18.38 (11.65) | .12 | .14 | 344 | 7.15 (3.03) | .74 | .032 |
| No | 156 | 38.83 (13.15) |  |  | 158 | 20.04 (8.10) |  |  | 154 | 26.72 (7.32) |  |  | 156 | 19.95 (10.38) |  |  | 157 | 7.24 (3.03) |  |  |
| **Population Centre Type** | | | | | | | | | | | | | | | | | | | | |
| Large | 556 | 39.06 (15.84) | .72 | .026 | 558 | 19.32 (8.62) | .071 | .129 | 557 | 25.41 (8.46) | **<.001^a^** | **.240** | 560 | 17.73 (11.54) | **.002^b^** | **.227** | 214 | 2.04 (0.16) | **<.001^c^** | **.325** |
| Small/Medium | 300 | 39.44 (13.70) |  |  | 303 | 20.42 (8.28) |  |  | 301 | 27.37 (7.60) |  |  | 300 | 20.31 (10.98) |  |  | 286 | 3.21 (0.19) |  |  |

^a^ In post-hoc testing using one-way ANOVA, there were significant differences in Toughness scores between youth in both large and medium population centres, and between youth in large and small population centres, but not between youth in medium and small population centres. Youth in small population centres reported the highest Toughness scores, followed by youth in medium population centres, and then youth in large population centres.

^b^ In post-hoc testing using one-way ANOVA, there was a significant difference in NTSM scores between youth in large and small population centres only. There were not statistically significant differences in NTSM scores between youth in large and medium population centres, or between youth in medium and small population centres.

^c^ In post-hoc testing using one-way ANOVA, there was a significant difference in HCA scores between youth in large and small population centres only. There were not statistically significant differences in HCA scores between youth in large and medium population centres, or between youth in medium and small population centres.

**Supplemental Table 3**

***Correlations with HCA Total Score by Ethnocultural Group***

|  | White Boys  (*n* = 348) | Ethnocultural Boys  (*n* = 196) |
| --- | --- | --- |
| Correlation (*r)* between HCA total score and… | | |
| MRNI-A-r: EDD | .22*** | .32** |
| MRNI-A-r: AF | .38*** | .25*** |
| MRNI-A-r: T | .28*** | .31*** |
| NTSM | .39*** | .21** |

**Supplemental Figure 1**

***Percentage of Cisgender, Heterosexual Participants who Indicate Any Form of Homophobic Name-Calling by Agent and Ethnocultural Group, 2016-2022 (n = 562)***

**Supplemental Figure 2**

***Percentage of Cisgender, Heterosexual Participants who Indicate Any Form of Homophobic Name-Calling by Agent and Population Centre Size, 2016-2022 (n = 562)***

*

*

*

*Participants who attended school in a small population centre were more likely to report any homophobic name-calling towards a friend (X^2^ (2, 506) = 10.79, *p* = .005) and someone they thought was gay (X^2^ (2, 505) = 12.89, *p*  = .002) than participants who attended school in a large population centre. Participants who attended school in a large population centre were less likely to use any homophobic name-calling towards someone they didn’t think was gay, as compared to participants who attended school in a medium or small population centre (X^2^ (2, 505) = 10.10, *p*  = .006).
